# Amplifying the voices of Black racial minorities in mental health research through public involvement and engagement: The importance of advisory roles

**DOI:** 10.1111/hex.13892

**Published:** 2023-11-20

**Authors:** Juliana Onwumere, Anthony Gentle, Rachel Obanubi, Annette Davis, Moffat Karuga, Rubbia Ali, BeFine Study Team, Valentina Cardi

**Affiliations:** ^1^ Department of Psychology, Institute of Psychiatry, Psychology and Neuroscience King's College London London UK; ^2^ South London and Maudsley NHS Foundation Trust London UK; ^3^ NIHR Biomedical Research Centre for Mental Health South London and Maudsley NHS London UK; ^4^ Department of Psychological Medicine, Institute of Psychiatry, Psychology and Neuroscience King's College London London UK; ^5^ Department of General Psychology University of Padova Padua Italy

**Keywords:** Black, health research, patient and public involvement and engagement

## Abstract

**Introduction:**

Ensuring adequate representation and the active, meaningful and visible involvement of groups likely to be most impacted by research findings and/or the lack of research inquiry are increasingly acknowledged. This is particularly relevant for Black racially minoritised groups who are less visible as research participants and in patient and public involvement and engagement (PPIE) roles. Our viewpoint article sought to discuss reflections and insights on their involvement experience, with particular attention to perceived barriers and enablers to PPIE involvement.

**Methods:**

Qualitative data were collected as part of facilitated group discussions from nine Black racially minoritised experts‐by‐experience involved in a PPIE advisory group. Data were subjected to thematic analysis to identify key themes.

**Results:**

Five main themes were identified that reflected factors linked to practicalities: role unfamiliarity, benefits for the larger community, acknowledgement of previous harm and mental health stigma.

**Conclusion:**

Given the existence and importance of the direct links between research and service and treatment innovations in health and social care, ensuring that those from underrepresented Black racial communities are meaningfully and equitably supported to have roles in advising and influencing research programmes should be prioritised and an ongoing consideration for different stakeholders, including research funders, researchers, healthcare providers and community leaders/representatives.

**Patient or Public Contribution:**

This viewpoint article is a collaboration between lived experience stakeholders and researchers, comprising conceiving the original idea for the paper, its conceptualisation and data generation and the coproduction including editing of the manuscript.

## 1 INTRODUCTION

Racial inequalities in different sections of society such as education, employment, wealth, criminal justice, healthcare and health outcomes are reported in different countries across the globe, including the United Kingdom (UK).^1^, ^2^, ^3^, ^4^, ^5^, ^6^, ^7^, ^8^, ^9^, ^10^ UK Black racially minoritised groups constitute approximately 4% of the population and typically comprise those from African and Caribbean ethnic backgrounds.^11^ Rates of mental health conditions are elevated in Black racially minoritised communities^12^ and they experience less positive care pathways.^13^, ^14^, ^15^, ^16^, ^17^, ^18^, ^19^ They are disproportionately affected by a broad range of social factors commonly associated with poorer mental health, including racism, unemployment, poverty and poorer housing,^4^, ^20^ and serve as barriers to volunteering.^21^

### 1.1 Links between research and healthcare

Innovations in health treatments including mental health, service design and delivery, which are targeted to the lived experience of service users and their families, largely reflect the areas prioritised for research funding and inquiry and are shaped by the perspectives of those consulted about and participating in research.^22^, ^23^, ^24^, ^25^ Consequently, the representation of and participation from racially minoritised groups in health‐related research, including opportunities to directly inform, shape and drive research priorities and research designs, form an important part of broader discussions on racialised health inequalities.^26^, ^27^, ^28^ Yet, our evidence‐based understanding of how best to implement a process that ensures the inclusion of groups disproportionately impacted by mental health problems and a broader range of racialised health and social inequalities is limited.^28^, ^29^, ^30^, ^31^

### 1.2 Health research involvement and racially minoritised groups

Despite a broader acknowledgement of the importance of having stakeholder involvement in the codesign and implementation of research studies, levels of engagement with and participation from Black racialised communities in health research are limited.^32^, ^33^ In some publications, Black communities continue to be described as ‘hard to reach’ or ‘hard to access’ and, in some cases, less interested in research.^34^ These descriptions, inadvertently, frame research involvement and inclusion as the responsibility of individual participants and/or the communities that they form part of. However, while Black racialised communities remain hypervisible in mental health user groups,^35^ they remain invisible (or absent) in power and decision‐making roles that can influence service development and innovation via research.^25^, ^36^ Moreover, the broader context of social inequalities and unique challenges that Black groups can encounter (e.g., anti‐Black racism) and perpetuate their marginalisation and exclusion from research continues to be neglected.^37^ Their underrepresentation inevitably raises the risk of erroneous assumptions and conclusions being made about their lived experiences and their needs, the impact (including benefits) of treatments and services and overlooking issues that might be unique to, or predominate, within their communities.

Factors underpinning the limited involvement of Black communities in research studies are manifold^34^ but typically coalesce around key issues of (mis) trust in the research process (including with those representing the face of the research), reported research aims and objectives and in what way (or assumed way) findings will be used.^38^, ^39^ Reports of mistrust have their origins in the intersection of racism, research and medical innovations, which have led to well‐known abuses and harm perpetrated against Black communities and with accounts between communities and across generations.^40^ These abuses, which exist alongside a myriad of social injustices (e.g., racism), omnipresent social media and 24‐h (including fake) news, serve only to further exacerbate mistrust in organisations, including those focused on research.^41^ For example, we know that young people from UK Black racialised communities report high levels of mistrust towards National Health Service (NHS) mental health services, deeming them unsafe to engage with.^1^ In a recent UK parliamentary report, almost two thirds of Black people (60%) surveyed believed that the NHS does not equally protect their health when compared to White British peers.^3^ Further, Black and minority ethnic groups were also more likely than White groups to report less trust in the NHS, public health and government scientists during the COVID 19 pandemic.^42^ Barriers to research participation that are also commonly shared across many different groups, irrespective of racial and ethnic background, include factors such as lack of awareness and understanding of research studies, socioeconomic and logistical factors, generalised (nontargeted) recruitment approaches and researchers' negative (and stereotypical) attitudes and biases.^27^, ^31^, ^40^, ^42^ However, people from Black and racially minoritised backgrounds, like other groups, are interested in participating in relevant research.^33^

Lived experience and stakeholder involvement during the research process, including initial opportunities to map ideas and set priorities for target areas for investigation, can enhance research quality and impact.^27^, ^31^ However, patient and public involvement and engagement (PPIE) is often characterised as being (or perceived to be) predominantly represented by stakeholders from White ethnic and/or higher socioeconomic backgrounds.^27^, ^31^, ^43^ The process of facilitating meaningful stakeholder engagement in mental health research lacks clarity, particularly as it applies to racially minoritised groups with a long history of harm and negative impact from research.^31^

Best practice recommendations for PPIE typically focus on trust‐building activities and targeted communications.^44^, ^45^ The extent to which these activities and approaches are developed and refined using a racialised and/or intersectional lens and directly informed by the needs and lived experiences of homogeneous racialised communities are unclear. Further, where research studies have sought to secure the involvement of and representation from racially minoritised groups, the calls to participate and data analyses and interpretation can often lack specificity and nuance and increase risk or perception of tokenism and/or lacking authenticity.^31^ The use of generic group descriptors, for example, ‘Black & Minorty Ethnic’ ‘Black, Asian & Minorty Ethnic’ also predominates and further contributes to a lack of specificity. Though laudable, generic calls for the inclusion of racially minoritised groups in research studies coupled with large generic group classifications can conceal the unique experiences of individuals from disparate racial backgrounds, which invariably shape their experiences, perspectives and health. To optimise overall engagement, representation and satisfaction in health research, developing opportunities that support PPIE from homogeneous racialised groups, which are also directly informed by homogeneous racialised communities, might serve as an important pathway forward.^46^ Moreover, efforts to understand the experiences of those who get involved might also offer helpful insights on how to improve representation.

In February 2021, our research group obtained funding from the United Kingdom Research Innovation Economic and Social Research Council for a study aimed at identifying and targeting the mental health needs of families from Black racial minority groups. The **B**lack **F**amilies **I**nvolvement in **N**ew **E**‐learning (BeFINE) project was aimed at answering three timely research questions among individuals from Black families: (1) How have families coped with COVID‐19 and its related challenges? (2) What types of support would they have found helpful over the course of the pandemic? (3) If available, what type(s) of support, resources and information would they find beneficial in their parental role in advancing the mental health and positive well‐being of their young children? The strategy to answer these questions was based on two crucial cornerstones: seeking the active and visible involvement of adults from Black racially minoritised groups in our stakeholder engagement and the use of mixed methodologies to explore different perspectives and lived experiences.

This viewpoint aimed to discuss qualitative feedback from BeFine PPIE members attending a stakeholder advisory group, with particular attention to perceived barriers and enablers to their involvement. This work represented a core and routine part of our public involvement and engagement activities and feedback exercises with members, and no ethical approval was required as part of our reflections. Nevertheless, all contributors to the advisory group were part of co‐producing and agreeing to standards of how the group would function and members engage with and communicate with one another. This included explicit reference to, and standards and expectations about personal disclosures, confidentiality, it being okay to not always have the right terms or language and conducting ourselves with an assumption of good intent from others. The work presented in this paper captures feedback gathered over the course of 10 advisory group meetings held between March 2021 and June 2022. See Tables 1, 2 for advisory group description and methods and analyses plan.

**Table 1:** Stakeholder advisory group.

Stakeholder advisory group
The BeFINE stakeholder advisory group comprised diverse members from Black racially minoritised communities in the United Kingdom and were attended by the projects coleads and facilitated by a research assistant. Members provided detailed input and feedback guiding important research decisions throughout all aspects of the research process. This ranged from selecting the project name and participating in an interview panel to select research assistants to work on the project, through to agreeing on the suitability of assessment materials and the identification and support of optimal participant recruitment strategies. Their contribution and feedback were also sought on the process and running of the stakeholder group to support their enhanced participation and ownership.
The stakeholder advisory group included nine adult members comprising a combination of those with their own lived experiences of mental healthcare, parenting and dual roles as parental caregivers in mental health. Members were evenly split in terms of their gender composition; predominantly self‐defined as coming from a Black Caribbean background (80%); and mostly in paid employment (80%), with age parameters falling between 20–30 and 50–70 years. Average attendance was approximately six members per meeting.

Abbreviation: BeFINE, **B**lack **F**amilies **I**nvolvement in **N**ew **E**‐learning.

**Table 2:** Methods and analyses plan.

Methods and analyses
Qualitative data in the form of transcripts from facilitated discussions, member reflections and feedback exercises on their involvement experience and perceived barriers and enablers to their involvement were subjected to a thematic analysis approach that was informed by Braun and Clarke^47^ to identify key themes. The analysis, led by the first author, began with a familiarisation process that included repeated reading of meeting transcripts and progressed with detailed review of each line of text. Initial codes were identified and organised around similarities and differences. Discussions were held with group members to discuss and agree on the final themes. All PPIE members were active in the process of reviewing and agreeing on final themes and highlighted areas. The research team, drawn from ethnically and racially minoritised backgrounds, including Black African, also held their own regular meetings to reflect on broader issues affecting racial minorities and their personal and professional implications and impacts.

Abbreviation: PPIE, patient and public involvement and engagement.

## 2 RESULTS: KEY THEMES

### 2.1 Practicalities

A key theme extracted from members' reports reflected the importance of attending to practical considerations to facilitate initial and continued involvement from members. Practical considerations were diverse but included having a regular meeting schedule of approximately every 6–8 weeks, the late evening start times, running meetings on the same day of the week (to support diary planning) and using an accessible video conferencing platform (Zoom), which many members had become familiar with over during the Covid‐19 pandemic and late evening meeting starts. The 20:00 GMT (i.e., late evening) scheduling was considered a preferable time and offered some flexibility to allow for completion of their various responsibilities such as home chores, childcare and employment, roles that were nonnegotiable and could not be relegated for an advisory role irrespective of its importance and relevance.

### 2.2 For the greater good

A second theme capturing a key factor underpinning their involvement and participation in the group related to perceived benefits to the wider Black community and playing an active role in achieving this. For example, reports of wanting to offer insights on the lived experiences of mental health problems; wanting to advance Black minoritised groups' involvement within health research; and wanting to meet with other community members and discuss pertinent issues related to mental ill health in Black communities with the purpose of improving community understanding of these issues were all shared. The relevance of the project and learning insights to ordinary people from everyday Black communities with families caring for children remained of central importance.

### 2.3 We are not used to this

The novelty of being in research and, specifically, being unaccustomed to serving in an advisory capacity was reflected in the third theme. Underpinning this was a shared belief amongst group members that the perspectives of a ‘Black person’ were rarely sought on matters of relevance, including in health research. Moreover, where views were collated, it was felt that their views were not necessarily given equal weight, relative to other groups. Therefore, this was felt to increase the likelihood of Black communities avoiding directly sharing their views on issues that had direct relevance to and implications for their contribution and involvement in the advisory group. The importance of researchers and participants, alike, not underestimating how novel the advisory roles were, was noted. There was an acknowledgement that where Black communities are invited to share their perspectives in a candid manner, such as the advisory group, and in the absence of it directly or indirectly leading to disadvantage for oneself and/or their peers, was a process that can take some time getting used to.

### 2.4 Acknowledging concerns

The group was united in their desire to access a project that from the outset, explicitly acknowledged their ongoing concerns and fears about trustworthiness and different harms that they (and/or the Black communities that they identified with) might have endured as part of research initiatives. Members welcomed their stakeholder roles as it provided a defined space where they felt their voices were valued. It offered opportunities to generate material to support change at a community (local) level, but where there was transparency about the process being followed by the research team and the different research stages, including the process of trying to secure ethical approval and pace of change.

### 2.5 Stigma

The power and impacts of mental health stigma and mental health illiteracy in Black communities are represented in the final theme. The need for researchers to acknowledge the additional challenges, efforts and decision‐making required by members before agreeing to being involved in a mental health‐related research and PPIE initiatives were highlighted. Group members were renumerated for their time and afforded a choice in what way they preferred to receive payment. However, members also fed back on their appetite for having more frequent and additional meetings without payment. It was felt that given a perceived lack of discussion, public disclosures and literacy about mental health issues in Black communities, opportunities to have a facilitated platform to meet with like‐minded others, within the advisory group, to focus on mental health matters was payment enough. See Table 3 for illustrative quotes.

**Table 3:** Key themes and illustrative quotes.

Key theme	Illustrative quote
Practicalities	*I like that the groups have been focused with an agenda and that we have all worked with as a group dynamic. I haven't felt over burdened with tasks to do and all those asked I have found those asked I have found those stimulating and enjoyable. I am glad….we are informed what we will discuss next so we can prepare ourselves for the group when we meet*.
For the greater good	*I wanted to see If I could help in any way I could and try and give something back to the community. I hoped that I would be able to use some of my experiences to maybe help and give input that might be helpful for carers and researchers….I believe the subject area was really important….in Black communities and I want to support this and that was also pivotal to me being involved in this advisory group*.
We are not used to this.	*Though I knew its purpose, I wasn't entirely sure what it would be like to be part of an advisory group….Its important that health research has the exposure necessary to encourage people from Black communities to have the opportunity to learn about have more involvement in projects like this*.
	*I appreciate that XXXXX prompts people to speak as it can be a daunting experience to speak up due to a lack of trust this community has in the institution of mental health…I appreciate the environment as I believe my voice can be heard*.
Acknowledging concerns	*Having this space is most helpful…. important to encourage community members to realise that their opinions are valid and appreciated and the hope is that a change can be made through this*.
	*It is important that these [groups] have people who resemble the people you are trying to cater for. There should be a mixture of Black people not just one sub‐group Caribbeans or Africans. We should be a mixture as we all come with our own similarities but also differences*.
Stigma	*It should be made clear as to where this research will go and there should be a space to reflect on whether people from this community would feel comfortable to discuss these topics*.
	*I have really enjoyed the discussions we have had. It has been welcome for me to be involved in discussions with others from a minority background because in my everyday life I do not get that much exposure to this opportunity*.

## 3 DISCUSSION

This project sought to examine the experiences of Black racially minoritised adults involved in a stakeholder advisory group for a mental health study. Their feedback yielded five themes, which suggested the importance of transparency and acknowledging how Black communities have historically been harmed by institutions involved in research and the negative impact on community trust. Trust plays an important role in healthcare including service uptake.^48^ Therefore, awareness of factors that have contributed to and shaped mistrust in Black communities and strategies to mitigate these in PPIE activities,^46^ are supported by our current findings.

Identified themes also included pragmatic issues to enable inclusion (e.g., evening meetings) and acknowledging how novel the involvement process was for members. Inequities in the process of volunteering exist; groups that might wish to participate might be prevented from doing so or must navigate several barriers that might make it harder to get involved. Black minoritised communities, when compared to White peers, are more likely to be in less secure and low‐paid employment.^6^ Advisory group members did not want to jeopardise their jobs or caregiving roles. Ensuring that PPIE activities are not incompatible with completing other life responsibilities (e.g., childcare) is indicated to facilitate, potentially, greater inclusion.

Establishing community stakeholder involvement, where inclusion and diversity are integral features, and voices of underrepresented and historically harmed minoritised groups in research are visible, is not an unattainable ideal in relation to Black communities in health research. It requires a partnership approach and commitment to, and skills in, power sharing. It also needs clear communications and congruence between what researchers say and what they do, since disparities elevate the risk of research(ers) being perceived as nontrustworthy. Black racial minority stakeholder involvement and research partnerships can work well, but from a starting platform of mutual respect, a commitment to listening and shared agreement that intended plans might not always go well. As a diverse group of members from Black lived experience communities and researchers, we think that these effective partnerships are achievable.

### 3.1 Limitations

This paper has limitations. First, it represents perspectives from a small group of participants involved in one PPIE initiative relating to one research project. It does not, and cannot, reflect experiences of all those from Black communities. Consequently, the authors caution against generalising the findings to all Black groups. Second, while the manuscript captures the collaboration, power inequities inherently present in PPIE activities and research mean that participants might not have felt comfortable to express their views, for example, as they specifically relate to the research team.

## 4 CONCLUSION

Research is a key facilitator in healthcare provision, healthcare solutions and service innovation. Ensuring the inclusion of historically underrepresented Black racially minoritised groups in research advisory and consulting roles should be of equal importance to encouraging and facilitating their inclusion as research participants. Their absence in either area only serves to maintain or perpetuate health inequalities.

## AUTHOR CONTRIBUTIONS

Anthony Gentle, Annette Davis, Moffat Karuga, Rubbia Ali, Valentina Cardi and Juliana Onwumere all contributed to data collection and analysis, paper conceptualisation and the drafting and editing of the paper. Juliana Onwumere had an overall lead role in this process.

## CONFLICT OF INTEREST STATEMENT

The authors declare no conflict of interest.

## Data Availability

Data sharing is not applicable to this article.
